# Research progress and prospect of postoperative adjuvant therapy for resectable intrahepatic cholangiocarcinoma

**DOI:** 10.3389/fphar.2024.1432603

**Published:** 2024-08-07

**Authors:** Yanxin Sun, Wei Jiang, Ruiheng Duan, Lianyue Guan

**Affiliations:** Department of Hepatobiliary-Pancreatic Surgery, China-Japan Union Hospital of Jilin University, Changchun, China

**Keywords:** intrahepatic cholangiocarcinoma, surgery, recurrence, adjuvant therapy, liver cancer, beneficiary population

## Abstract

Intrahepatic cholangiocarcinoma (ICC) is the second most common primary malignancy of the liver, following hepatocellular carcinoma (HCC). Surgical resection remains the only potentially curative treatment for ICC. However, due to its high malignancy and propensity for postoperative recurrence, the prognosis for ICC is generally poor, and there is currently little standardized approach for adjuvant therapy following curative surgery. This article aims to explore adjuvant treatment strategies for ICC post-curative surgery by reviewing retrospective studies and clinical trials conducted in recent years. The analysis focuses on the effectiveness, challenges, and potential developments in the management of ICC post-surgery, considering the high recurrence rates and the need for improved therapeutic approaches to enhance patient outcomes. Additionally, we discuss the various types of adjuvant treatments that have been explored, including chemotherapy, radiation therapy, and targeted therapies. The goal is to provide a comprehensive overview of the current landscape and highlight promising directions for future research to improve survival and quality of life for ICC patients.

## 1 Introduction

**FIGURE 1:**
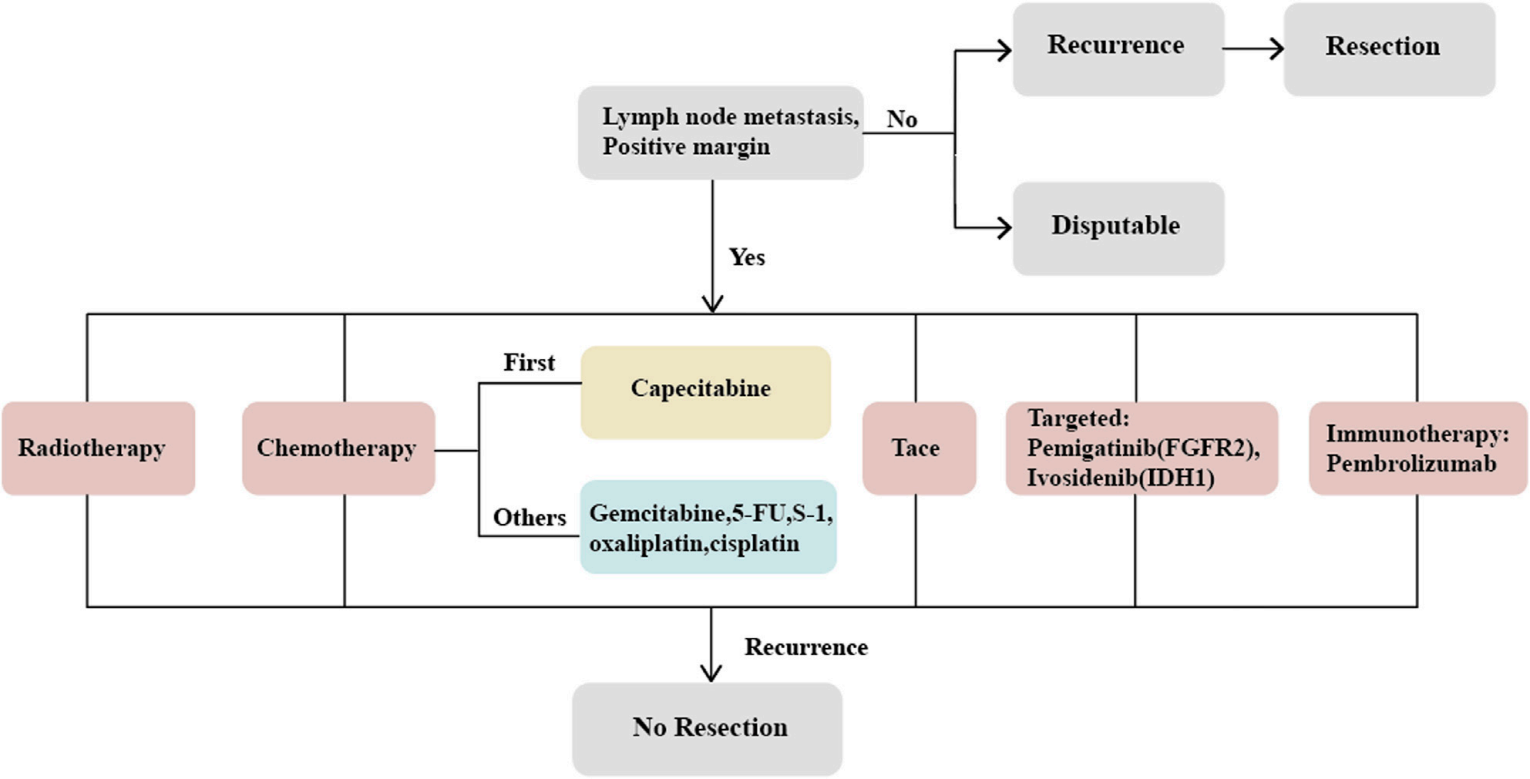
The logical flow of the article.

Cholangiocarcinoma (CCA) is a highly invasive malignancy originating from the epithelial cells of the bile ducts, with a 5-year survival rate of less than 20% (Lamarca et al., 2022). Based on the anatomical site of occurrence, CCA can be classified into intrahepatic cholangiocarcinoma (ICC) and extrahepatic cholangiocarcinoma (ECC). ICC accounts for only 10%–20% of all CCA cases, yet its incidence has been rising globally in recent years (Valle et al., 2016; Sirica et al., 2019; Sung et al., 2021; Valle et al., 2021), with significantly higher rates in Asian populations compared to Western countries (Chinese Society of Liver Cancer Cholangiocarcinoma Cooperative Group, 2022). ICC originates from epithelial cells within the hepatic bile ducts and is the second most common primary liver cancer following hepatocellular carcinoma (HCC), comprising 10%–15% of primary liver cancers (Massarweh and El-Serag, 2017; Bray et al., 2018; Bertuccio et al., 2019; Li Y. et al., 2022). Despite its rarity (Machairas et al., 2020), ICC predominantly affects individuals between 50 and 70 years of age and is less common in those under 40 (Bertuccio et al., 2019). Sporadic cases of ICC predominate, but certain risk factors have been identified, including parasitic infections, primary sclerosing cholangitis (PSC), congenital bile duct abnormalities, intra- and extra-hepatic cholelithiasis, hepatitis B and C virus infections, cirrhosis, non-alcoholic fatty liver disease, diabetes, alcohol consumption, smoking, obesity, and exposure to environmental chemicals (Petrick et al., 2018; Makiuchi et al., 2019; Clements et al., 2020; De Lorenzo et al., 2020; Elvevi et al., 2022). The incidence of ICC is closely related to the geographical region, with PSC being the most common etiology in Western countries, where 5%–10% of PSC patients eventually develop ICC (Brindley et al., 2021; Catanzaro et al., 2023); in Southeast Asia, which has the highest incidence, parasitic infection is the predominant cause (Shen and Shen, 2021). Nonetheless, approximately 50% of ICC cases have no identifiable cause (Kelley et al., 2020), leading to a delayed diagnosis and loss of therapeutic opportunity. The prognosis for ICC patients is very poor; curative surgery is the only effective treatment, yet only 20% of patients are eligible for curative resection at the time of diagnosis. Even after curative surgery, the recurrence rate for ICC can be as high as 60%–70%, resulting in a 5-year survival rate of only 30%–40%. Patients with positive lymph nodes fare even worse, with survival rates around 20%, underscoring the urgent need for effective adjuvant therapies to control tumor progression postoperatively (de Jong et al., 2011; Amini et al., 2014; Bridgewater et al., 2014; Squires et al., 2018; Cillo et al., 2019; Chinese Society of Liver Cancer Cholangiocarcinoma Cooperative Group, 2022; Lamarca et al., 2022). However, the benefit of adjuvant therapy remains highly controversial, and currently, there is no consensus on the optimal adjuvant treatment strategy (Luvira et al., 2021; Elvevi et al., 2022; Krenzien et al., 2022). Research specifically targeting adjuvant therapy for ICC postoperatively is scant (Krenzien et al., 2022). The PRODIGE trial, a multicenter phase III clinical study including CCA and gallbladder carcinoma (GBC), compared the GEMOX chemotherapy regimen with observation. The final results showed no significant difference in relapse-free survival (RFS) or overall survival (OS) between the two groups (Edeline et al., 2019). In contrast, a separate phase III study demonstrated the efficacy of capecitabine (Primrose et al., 2019), providing the theoretical basis for the latest guidelines by the National Comprehensive Cancer Network (NCCN) and the American Society of Clinical Oncology (ASCO), which recommend capecitabine as the new standard chemotherapy post-biliary surgery (Shroff et al., 2019). In recent years, with the diversified development of treatments for gastrointestinal tumors, a multitude of therapeutic approaches such as chemotherapy, radiotherapy, local treatments, targeted therapies, and immunotherapy, used either alone or in combination, are contending for predominance. This article will discuss the adjuvant treatment strategies for ICC post-curative surgery in light of the current treatment modalities for CCA (Figure 1).

## 2 Beneficiary population for adjuvant therapy

The determination of whether all patients with intrahepatic cholangiocarcinoma (ICC) require adjuvant therapy following hepatic resection remains a subject of ongoing debate. However, there is a consensus within the medical community regarding the necessity of adjuvant therapy for patients with positive lymph nodes and positive surgical margins after resection for ICC (Miura et al., 2015; Sutherland et al., 2022). The research conducted by Sheka et al. (2020), Martin et al. (2020), and Altman et al. (2020) has collectively affirmed that patients with nodal metastases derive significant benefit from adjuvant treatments post-curative surgery. Furthermore, Littau et al. (2022) have demonstrated that the 5-year survival rates for patients with R0 resection are superior to those with R1 resection, indicating a heightened need for adjuvant therapy in R1 resected patients. This position is corroborated by earlier meta-analytic findings (Horgan et al., 2012). Subsequent investigations have identified additional determinants of recurrence in ICC following curative interventions, such as the number of tumors, as reported by Buettner et al. (2019). A meta-analysis by Song S. et al. (2022) revealed that patients with vascular invasion notably benefit from postoperative chemotherapy. Research by Wei et al. (2022) correlated neural invasion with poorer prognostic outcomes, and elevated levels of carbohydrate antigen (CA) 19-9 were linked to unfavorable prognoses in the studies by Kim et al. (2018) and Pavicevic et al. (2022). Moreover, Ma et al. (2016) indicated that a tumor margin of less than 1 cm constitutes a high-risk factor for recurrence. Compiling existing literature, it emerges that the high-risk factors for postoperative recurrence of ICC include lymph node metastasis, positive surgical margins, the presence of multiple tumors, low tumor differentiation, tumor size exceeding 5 cm, vascular and neural invasion, elevated CA 19-9 levels, and narrow resection margins of less than 1 cm (Kim et al., 2018; Song Y. et al., 2022; Littau et al., 2022; Suzuki et al., 2022; Wei et al., 2022). Consequently, ICC patients exhibiting these risk factors should be the focus of vigilant postoperative management. Additionally, studies suggest that high expressions of CYFRA 21-1 and IL-6 in tumor tissue (Huang et al., 2015), IL-17 surrounding tumor cells (Asukai et al., 2015), and the genes SDHAF2, MRPS34, MRPL11, and COX8A in cancer stem cells (Huang et al., 2022) are associated with a poor prognosis, thus offering novel prognostic avenues for exploration. According to the 8th edition guidelines of the American Joint Committee on Cancer (AJCC), the utility of adjuvant therapy in stage I ICC patients without high-risk factors remains contentious (Lee and Chun, 2018); however, a more recent retrospective study from China has indicated that stage I patients may derive greater benefits from adjuvant therapy following curative resection than those with more advanced disease (Su et al., 2022). Moreover, another meta-analysis has also concluded that adjuvant therapy postoperatively can indeed be beneficial for ICC patients, although further studies are necessary to identify the specific subgroups most likely to benefit (Ke et al., 2020).

## 3 Adjuvant treatment strategies

The high recurrence rate of intrahepatic cholangiocarcinoma (ICC) post-surgery underscores the imperative for effective postoperative intervention (Gkika et al., 2020). A recent comprehensive meta-analysis has demonstrated that adjuvant therapies, including chemotherapy (CT), radiotherapy (RT), and transcatheter arterial chemoembolization (TACE), contribute beneficially to patients’ relapse-free survival (RFS) and overall survival (OS) (Ke et al., 2020). Furthermore, the emergence of targeted immunotherapies has provided additional therapeutic support for patients following ICC surgery.

### 3.1 Chemotherapy

Although most retrospective studies have confirmed the benefit of adjuvant chemotherapy for patients with high-risk ICC in improving overall survival (OS) and relapse-free survival (RFS), there is still no consensus on the chemotherapy regimen following ICC hepatic resection (Song Y. et al., 2022). Given the interim results of the BILCAP trial, adjuvant capecitabine has become the recommended drug for postoperative adjuvant treatment of both ICC and CCA (Primrose et al., 2019), although these advantages were not observed in the intention-to-treat analysis. Some studies suggest considerable benefits of postoperative chemotherapy, reducing the risk of death by up to 40%, but the choice of chemotherapeutic agents requires further prospective studies (Ghidini et al., 2017). The lack of standardized chemotherapy protocols for biliary tract malignancies results in varied regimens across institutions, including gemcitabine, capecitabine, 5-fluorouracil (5-FU), S-1, oxaliplatin, and cisplatin, either as monotherapy or in combination. A retrospective study with gemcitabine as the main chemotherapeutic agent indicated that adjuvant chemotherapy is beneficial for ICC patients with intrahepatic bile duct stones (Li Q. et al., 2022); however, a meta-analysis pointed out that adjuvant gemcitabine therapy is favorable for patient prognosis post-ICC surgery, whereas 5-FU was not associated with prognosis (Ma et al., 2019; Ke et al., 2020). An early trial also showed that the combination of mitomycin C (MMC) with 5-FU did not exhibit efficacy in postoperative cholangiocarcinoma patients (Takada et al., 2002). In contrast, another meta-analysis highlighted that CT based on fluorouracil significantly improved OS in CCA patients, whereas gemcitabine-based CT did not demonstrate a clear benefit (Ma et al., 2020). Studies by Tran Cao et al. (2018) and Edeline et al. (2022) also concurred that gemcitabine monotherapy is not beneficial for a patient’s post-curative surgery for bile duct cancer. Nonetheless, Song S. et al. (2022) reported that postoperative use of 5-FU benefits patient prognosis and can serve as an adjuvant treatment for cholangiocarcinoma. The ABC-02 trial confirmed the role of gemcitabine combined with cisplatin in patients with advanced bile duct cancer, providing a new avenue for gemcitabine in adjuvant chemotherapy, but the efficacy of adjuvant treatment post-CCA resection still requires further substantiation. The ongoing ACTICCA-1 trial (NCT02170090) is recruiting post-resection CCA patients to compare gemcitabine combined with cisplatin versus capecitabine (Stein et al., 2015). Another ongoing phase III trials include Japan’s COG1202 (ASCOT) trial, which investigates the effect of S-1 on patients with BTC following surgery (Nakachi et al., 2018), and a multicenter trial in Korea (NCT04401709) comparing the efficacy of gemcitabine combined with capecitabine to monotherapy with capecitabine.

### 3.2 Radiotherapy

Currently, limited data are available to support the use of adjuvant radiotherapy following resection for intrahepatic cholangiocarcinoma (ICC) (Turgeon and Maithel, 2020). There have been no phase III clinical trials to evaluate the role of adjuvant radiotherapy (Valle et al., 2021), and it is most often combined with chemotherapy. However, due to the high local recurrence rate post-surgery for ICC, adjuvant radiotherapy has been employed in treating these patients (Yoo et al., 2021). An early retrospective study by Shinohara et al. (2008) demonstrated a significant advantage of postoperative adjuvant radiotherapy over surgery alone. Furthermore, research by Lin et al. (2018) indicated that adjuvant radio chemotherapy post-ICC surgery outperformed chemotherapy alone, especially for patients with positive surgical margins and advanced AJCC stages (III and IV). Dee et al. (2020) study also found that postoperative adjuvant radio chemotherapy aided in improving the OS and RFS for patients with positive margins. Conversely, Hammad et al. (2016) after analyzing data from 2,897 patients in the National Cancer Database, found that postoperative adjuvant radiotherapy did not benefit ICC patients, even those with positive margins; similarly, Tran Cao et al. (2018)’s study did not observe benefits of radiotherapy in patients with positive lymph nodes. Hammad et al. (2016)’s research indicated that postoperative adjuvant radiotherapy was only beneficial for patients with negative surgical margins. Given the controversy regarding postoperative radiotherapy for ICC, ASCO guidelines currently do not recommend adjuvant radiotherapy for postoperative ICC patients (Shroff et al., 2019). In contrast, the American Society for Therapeutic Radiology and Oncology (ASTRO) recommends external beam radiation therapy for ICC patients with high-risk features (Apisarnthanarax et al., 2022).

### 3.3 Transcatheter arterial chemoembolization

While the efficacy and safety of transcatheter arterial chemoembolization (TACE) have been established in patients with advanced, unresectable intrahepatic cholangiocarcinoma (ICC), its benefits for patients post-hepatectomy remain unclear (Chen et al., 2022). Recent meta-analyses on the effectiveness of adjuvant TACE postoperatively have yielded conflicting conclusions (Ma et al., 2019; Liu et al., 2020). A retrospective study by Shen et al. (2011), including 125 patients post-ICC resection, found that TACE was beneficial for patients with early recurrence and those presenting with multiple high-risk factors for recurrence, aligning with findings from Wu (Zhang et al., 2022), Li (Li et al., 2015), Lu (Lu et al., 2017), Cheng (Cheng et al., 2021), and others. Contrarily, Wang et al. posited that adjuvant TACE post-surgery benefits only patients with TNM stages II and III, suggesting that the more risk factors present, the higher the likelihood of recurrence, thus necessitating a more optimized adjuvant treatment strategy (Wang et al., 2020). These risk factors include CA19-9>37U/L, lymph node metastasis, tumor size>5 cm, and satellite. The article further points out that when the risk factors are ≤2, the effect is better, and when the risk factors are>2, other adjuvant treatment options such as radiotherapy and chemotherapy are needed. Moreover, another study demonstrated that patients with hepatitis B virus-related liver disease significantly benefited from postoperative adjuvant TACE (Jeong et al., 2017); however, Liu et al. (2021) indicated that for patients at TNM stage I, postoperative adjuvant TACE was not only unbeneficial but could potentially promote tumor recurrence. As for the drug selection for TACE treatment, there are no clear guidelines yet. Currently, epirubicin, 5-FU, oxaliplatin, etc. Can all be used as adjunctive drugs for selection.

### 3.4 Targeted and immunotherapy

In recent years, with a clearer understanding of the molecular aspects of diseases, intrahepatic cholangiocarcinoma (ICC) has entered a new era of targeted therapy. Two molecular targets, FGFR2 and IDH1/2, are particularly significant in ICC patients, with mutation frequencies of approximately 15%–18% and 10%–15%, respectively (Rizzo et al., 2021a; Javle et al., 2021; Komuta, 2021). Pemigatinib, a targeted drug for FGFR2, has passed the FIGHT 202 Phase II clinical trial and has been approved as a second-line treatment for ICC (Abou-Alfa et al., 2020). Additionally, the FIGHT 302 trial, investigating pemigatinib as a first-line treatment, is currently recruiting patients (NCT03656536), and other clinical trials for FGFR2-targeted drugs are ongoing (Makawita et al., 2020). Furthermore, the IDH1 inhibitor ivosidenib, due to its encouraging performance in the ClarIDHy trial, has also been approved as a second-line treatment for ICC (Zhu et al., 2021). Research is underway on other therapeutic targets as well, although the lower mutation rates pose greater challenges for study.

Immunotherapy, emerging alongside targeted therapy as a novel treatment modality, has shown promising results in hepatocellular carcinoma (HCC) but is still primarily in the clinical trial phase for intrahepatic cholangiocarcinoma (ICC) (Rizzo et al., 2021b). Pembrolizumab, used in the immunotherapy of advanced biliary cancer, has been featured in multiple clinical trials and has yielded favorable outcomes. Additionally, research has indicated that the combination of nivolumab with gemcitabine and cisplatin has achieved positive results, paving the way for the combined application of various treatment modalities.

Overall, current targeted and immunotherapy approaches are primarily focused on patients with advanced stages of intrahepatic cholangiocarcinoma (ICC), with limited research dedicated to their use in postoperative adjuvant treatment. However, it is becoming increasingly evident that these therapies will play a vital role in the future of adjuvant treatment for ICC patients. The treatment of this disease is expected to evolve towards addressing it at the molecular level, which could lead to more effective and personalized therapeutic strategies.

### 3.5 Recurrence and subsequent resection

Intrahepatic recurrence is the most common form of relapse for intrahepatic cholangiocarcinoma (ICC) (Song Y. et al., 2022). Recent studies have shown that resection after recurrence can significantly extend patients’ overall survival (OS), sometimes achieving outcomes comparable to initial resections. However, these reports further indicate that patients with lymph node metastasis identified during the initial resection experience less favorable outcomes upon re-resection (Kitano et al., 2020). A multicentric retrospective study from Germany, which analyzed 113 patients undergoing surgical resection for recurrent ICC, reported a median overall survival of 65.2 months after repeat resection, with 1-year, 3-year, and 5-year survival rates of 98%, 78%, and 57%, respectively (Bartsch et al., 2021). These benefits have also been confirmed in other smaller-scale retrospective studies (Takahashi et al., 2015; Bartsch et al., 2019; Sakata et al., 2021), underscoring the potential value of re-resection as a therapeutic option in certain cases of recurrent ICC.

## 4 Discussion

Cholangiocarcinoma (CCA) is a rare and highly malignant tumor, presenting challenges in conducting randomized controlled trials due to difficulties in recruiting enough patients. This issue is compounded by two significant problems: first, many patients are diagnosed at a late stage, making them ineligible for surgical treatment and thus precluding subsequent adjuvant therapy; second, many trials encompass all types of biliary malignancies, which, given their heterogeneity depending on the location, necessitate a nuanced interpretation of the data and findings (Mazzaferro et al., 2020). This is also one of the reasons why many current studies cannot obtain the same or similar conclusions. Addressing the first problem, neoadjuvant therapy, which is increasingly researched, may offer hope for many patients with advanced stages, potentially downstaging their disease to make surgery feasible. However, current studies on neoadjuvant therapy for ICC are predominantly retrospective, and no guidelines explicitly advocate for its benefits (Fong et al., 2021). A phase II trial combining gemcitabine, capecitabine, and albumin-bound paclitaxel (Clinical Trials gov identifier: NCT03579771) is currently recruiting late-stage CCA patients. Regarding the second problem, there is a need to strengthen guideline development and consensus-building, enabling clinicians to more precisely classify cholangiocarcinoma patients and establish robust database systems for gathering higher-level evidence. A phase III trial in China (NCT02548195) is ongoing, comparing GEMOX with capecitabine for post-resection ICC patients.

Given the high recurrence rate post-surgery for ICC, many guidelines, and publications support adjuvant therapy for post-resection ICC patients (Fong et al., 2021). A review of published literature reveals that patients with positive lymph nodes and positive surgical margins are ideal candidates for adjuvant therapy. In a study by Sheka et al. (2020) involving 2,837 postoperative patients with lymph node positivity, the OS and RFS of surgery combined with adjuvant chemotherapy were significantly higher than those of simple surgery patients; In a study by Littau et al. (2022) involving 3,618 surgical resection patients, R1 resection of OS and RFS resulted in poorer prognosis and greater need for adjuvant treatment. Other factors warranting consideration for adjuvant treatment include multifocal tumors, low differentiation, tumor size greater than 5 cm, vascular invasion, neural invasion, and high levels of CA19-9. Studies have also shown that patients with low to moderate risk can benefit from adjuvant therapy, achieving better relapse-free survival (RFS) and overall survival (OS). Additionally, research indicates that factors such as high expression of CYFRA 21-1, IL-6, IL-17, and genes like SDHAF2, MRPS34, MRPL11, COX8A, and genetic mutations in TP53, KRAS, and CDKN2A are associated with poor prognosis in ICC (Boerner et al., 2021), suggesting new directions for adjuvant therapy. Current guidelines, based on the results of the BILCAP trial, recommend 6 months of capecitabine chemotherapy for all patients after curative surgery, providing a valuable reference for clinical practice.

Regarding the strategies for postoperative adjuvant treatment, the uncertainty surrounding radiotherapy and the developmental stage of targeted, and immunotherapy mean that chemotherapy currently stands as the mainstay of adjuvant treatment, especially for patients with high-risk factors, supplemented by transcatheter arterial chemoembolization (TACE). However, due to varying results from phase III clinical trials and retrospective studies, a standardized chemotherapy regimen has yet to be established. Drugs like capecitabine, gemcitabine, cisplatin, 5-fluorouracil (5-FU), and S-1 may all be beneficial to prognosis, but further randomized controlled trials and prospective studies are needed. The BILCAP III trial (Primrose et al., 2019) indicates that capecitabine is beneficial for OS and RFS in patients after biliary surgery, which is currently the latest evidence-based medicine evidence. The use of radiotherapy post-ICC surgery remains controversial, necessitating careful consideration. For patients at high risk of recurrence, adjuvant TACE represents an important component of the combined treatment approaches, although its benefits for early-stage patients post-surgery still require further validation. In addition, for patients with>2 high-risk factors, TACE alone cannot achieve good results, and multiple treatment methods such as combined radiotherapy and chemotherapy are needed (Wang et al., 2020). Research on targeted immunotherapies is relatively limited and primarily focuses on unresectable, advanced-stage patients. While the outcomes have been mixed, there is evidence suggesting that patients with specific genetic mutations might benefit from targeted therapies. For example, the targeted drugs Pemigatinib and Ivosidenib for FGFR2 and IDH1, which have relatively high mutation rates, have become second-line treatment drugs for advanced ICC. Studies have also indicated that immunotherapy can enhance the sensitivity of cancer cells to chemotherapy, offering a promising direction for combination therapy post-surgery. Additionally, in cases where ICC recurrence is confined to the liver, repeat resection could be a favorable option.

## 5 Conclusion

In summary, given the rarity, high malignancy, and heterogeneity of intrahepatic cholangiocarcinoma (ICC), randomized controlled trials are scarce for postoperative adjuvant therapy, resulting in the lack of a standardized treatment protocol. Therefore, I urge clinicians to diligently follow up with ICC patients to gather more data, which could contribute to higher-level evidence. Additionally, as medical science advances and our understanding of the molecular aspects of diseases becomes clearer, this knowledge may provide more effective treatment methods for ICC in the future. The focus on molecular-level research is likely to lead to more targeted and personalized therapeutic approaches, potentially improving outcomes for patients with this challenging and complex disease.

## Abbreviations

5-FU, 5-Fluorouracil; AJCC, American Joint Committee on Cancer; ASCO, American Society of Clinical Oncology; ASTRO, American Society for Therapeutic Radiology and Oncology; CA, Carbohydrate Antigen; CCA, Cholangiocarcinoma; CT, Chemotherapy; ECC, Extrahepatic Cholangiocarcinoma; GBC, Gallbladder Carcinoma; HCC, hepatocellular carcinoma; ICC, Intrahepatic cholangiocarcinoma; MMC, Mitomycin C; NCCN, National Comprehensive Cancer Network; OS, Overall Survival; PSC, Primary Sclerosing Cholangitis; RFS, Relapse-Free Survival; RT, Radiotherapy; TACE, Transcatheter Arterial Chemoembolization.
